# Dendritic cell vaccination with a toll-like receptor agonist derived from mycobacteria enhances anti-tumor immunity

**DOI:** 10.18632/oncotarget.5281

**Published:** 2015-09-16

**Authors:** Manh-Cuong Vo, Hyun-Ju Lee, Jong-Seok Kim, My-Dung Hoang, Nu-Ri Choi, Joon Haeng Rhee, Vinoth-Kumar Lakshmanan, Sung-Jae Shin, Je-Jung Lee

**Affiliations:** ^1^ Research Center for Cancer Immunotherapy, Chonnam National University Hwasun Hospital, Jeollanamdo, Republic of Korea; ^2^ Department of Hematology-Oncology, Chonnam National University Hwasun Hospital, Jeollanamdo, Republic of Korea; ^3^ Department of Microbiology and Institute of Immunology and Immunological Diseases, Yonsei University, Seoul, Republic of Korea; ^4^ Department of Microbiology, Chonnam National University Medical School, Gwangju, Republic of Korea; ^5^ Department of Biomedical Science, Chonnam National University Medical School, Gwangju, Republic of Korea

**Keywords:** dendritic cells, *mycobacterial* heat shock protein 90, mouse colon cancer

## Abstract

Dendritic cell (DC)-based vaccines are considered useful in cancer immunotherapy, and the interaction of DC and adjuvants is important in the design of the next generation vaccines. In this study, whether DC combined with Rv2299c derived from mycobacteria could improve anti-tumor immune responses in a colon cancer mouse model was evaluated. MC38 cell lines were injected subcutaneously to establish colon-cancer-bearing mice and the following four groups were evaluated: PBS control, tumor antigen (TA) loaded-DC, Rv2299c, and a combination of TA-loaded-DC and Rv2299c. The combination treatment with TA-loaded-DC and Rv2299c exhibited greater inhibition of tumor growth compared to other groups. These effects were associated with the reduction of suppressor cells, such as myeloid-derived suppressor cells and regulatory T cells, and the induction of effector cells, such as CD4^+^ T cells and CD8^+^ T cells, in spleen, and with the activation of cytotoxic T Lymphocytes and NK cells. These results suggest that TA-loaded-DC vaccination with Rv2299c derived from mycobacteria enhanced anti-tumor immunity in a mouse colon cancer model by inhibiting the generation of immune-suppressive cells and recovering numbers of effector cells, and demonstrated superior polarization of the Th1/Th2 balance in favor of the Th1 immune response.

## INTRODUCTION

Immune-based therapeutic options that use antigen-presenting cells (APCs) with increased potency are considered an attractive tool in cancer immunotherapy [1–4]. Dendritic cells (DC), the most potent APCs, play a central role in various immunotherapy protocols via the generation of cytotoxic T lymphocytes (CTLs) [5, 6]. Tumor antigen (TA)-specific immunotherapy is an emerging approach in cancer treatment. Most TAs are self-proteins, thus, there is a need to employ vaccine adjuvants that can stimulate the efficient presentation of weakly immunogenic proteins in a manner that allows effective activation of T cells [7]. Potent adjuvants are prerequisites to the immunotherapy for overcoming low immunogenicity of TA [8]. Substantial progress has been made in identifying adjuvants that recruit and/or activate appropriate APCs to elicit tumor-specific immunity [9]. The use of a cocktail of toll-like receptor (TLR) ligand agonists as adjuvants in an anti-viral vaccine significantly enhanced the functional avidity of the T cells, rather than increasing their numbers, markedly improving the anti-viral response [10]. Intrinsic recognition of the TLR ligands induces maturation of APCs [11].

Heat shock protein 90 (HSP90) has adjuvant activity via association with TLR4 signaling in innate immune cells [12, 13]. The interaction of HSP-peptide complexes with APCs leads to the presentation of antigenic peptides to CD8^+^ and CD4^+^ T cells and to a cascade of non-antigen-specific events that activate APCs and promote immune responses [14]. Activation of the innate immune system by HSP90 induced various effects on *in vivo* tumor immunogenicity in experimental animal models [15–18]. *Mycobacterium tuberculosis* Rv2299c is a member of the HSP90 family [19, 20]. Rv2299c is similar to the HSP high-temperature protein G (htpG) homologue from *Bacillus subtilis* [19, 20]. It is possible that in a tuberculous granuloma both pathogens and host cells are stressed, leading to the production HSP.

In this study, whether the combination of DC with Rv2299c as an adjuvant resulted in enhanced activation of DC was evaluated. In theory, large numbers of activated DC would prime highly functional tumor-specific T cells to higher levels, enhancing the clinical efficacy of adjuvant vaccines. This study demonstrated that TA-loaded-DC vaccination with Rv2299c derived from mycobacteria enhanced anti-tumor immunity in a mouse colon cancer model by inhibiting immune-suppressive cells and recovering effector cells, and demonstrated superior polarization of the Th1/Th2 balance in favor of the Th1 immune response.

## RESULTS

### BM-derived CD11c^+^ DC showed a fully mature phenotype and marked IL-12p70 secretion

**Figure 1:**
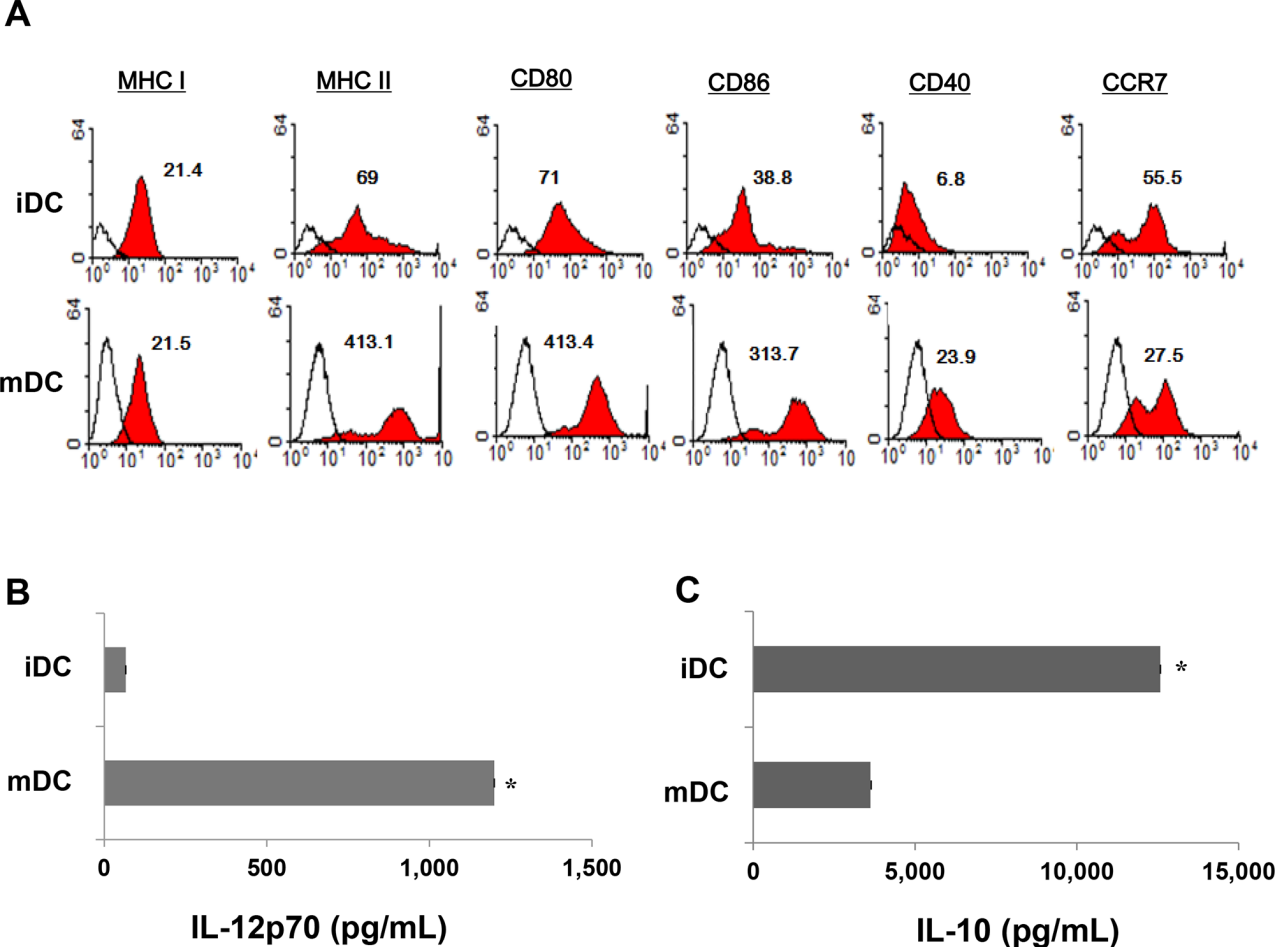
Characteristics of bone-marrow-derived CD11c^+^ dendritic cells (DC) **A.** The phenotype of DC were analyzed for expression levels of MHC I, MHC-II, CD80, CD86, CD40, and CCR7 using flow cytometry; mature DC (mDC) expressed higher levels of maturation molecules than immature DC (iDC). Representative histograms show marker expression levels (shaded) compared to those of the isotype controls (black line). **B, C.** An ELISA revealed that mDC produced higher levels of IL-12p70 and lower levels of IL-10 in culture supernatants after subsequent CD40L stimulation, compared to iDC (*P* < 0.05). The data are shown as the means (pg/mL) ± standard deviation (SD) of triplicate cultures from three independent experiments.

BM-derived CD11c^+^ DC were maturated with GM-CSF, TNF-α, and IL-1β, and then loaded with gamma-irradiated MC-38 cells, which constituted ∼96% of cells that underwent apoptosis (Supplemental Figure 1). The DC expressed higher levels of several molecules related to DC maturation (Figure 1A) than iDC and produced higher levels of IL-12p70 (Figure 1B) and lower levels of IL-10 (Figure 1C) after subsequent CD40L stimulation compared to iDC.

### Rv2299c anti-tumor immunity effects in a colon cancer mouse model

**Figure 2:**
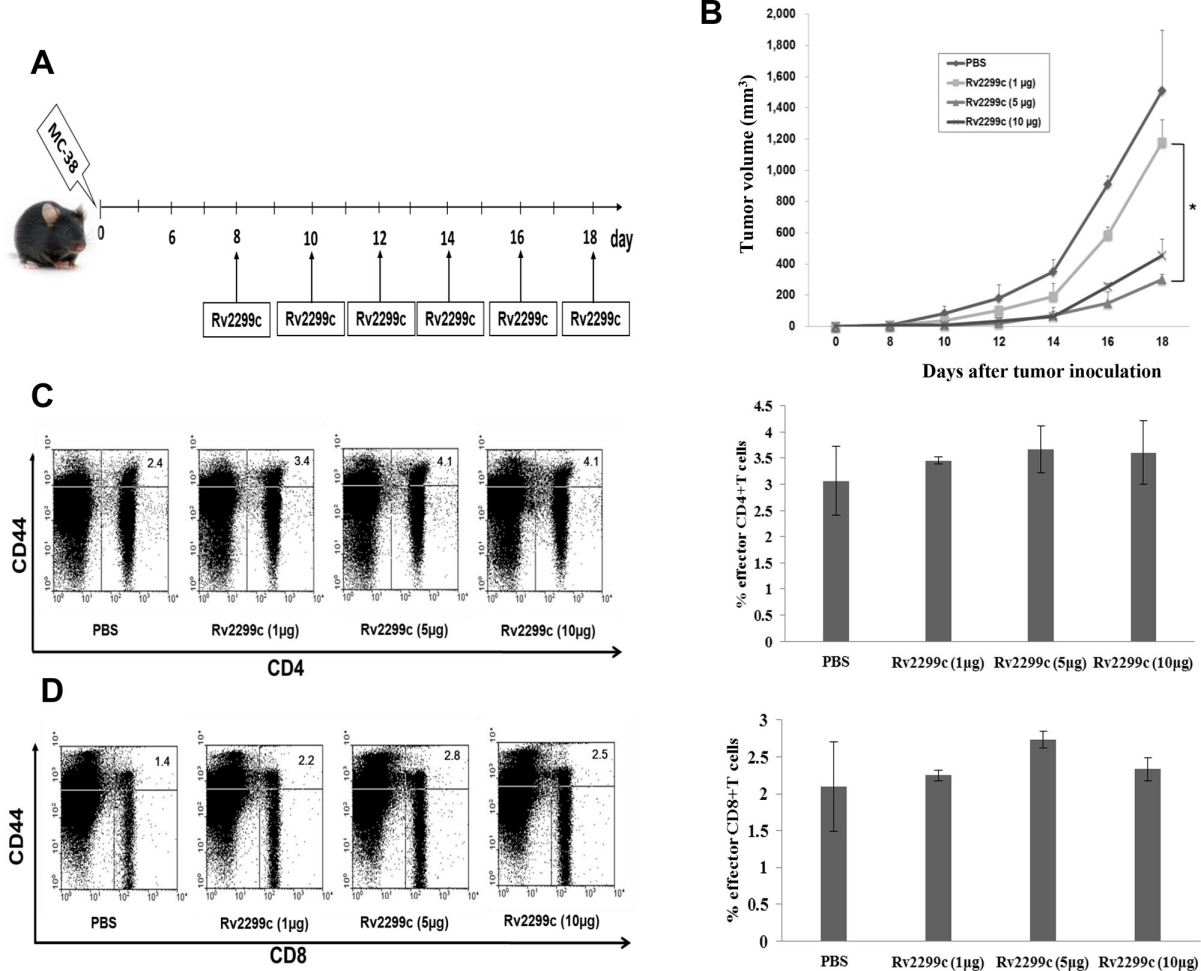
Rv2299c injection results in enhanced anti-tumor activity against MC-38 colon cancer **A.** Schematic experimental schedule of *in vivo* animal vaccinations with Rv2299c. Experiments consisted of five mice per group. **B.** Mice treated with 5 and 10 μg of Rv2299c showed significantly greater inhibition of tumor growth compared to those receiving 1 μg of Rv2299c or the PBS control (*P < 0.05), as measured by the monitoring tumor volume. **C, D.** Mice in the 5- and 10-μg Rv2299c treatment groups had an increased proportion of CD4^+^ T cells and CD8^+^ T cells among splenocytes compared to the 1-μg Rv2299c or PBS control group, as measured by flow cytometry in the left panel and compared by quantitated bar graphs in the right panel. Data are representative of more than 3 experiments.

**Figure 3:**
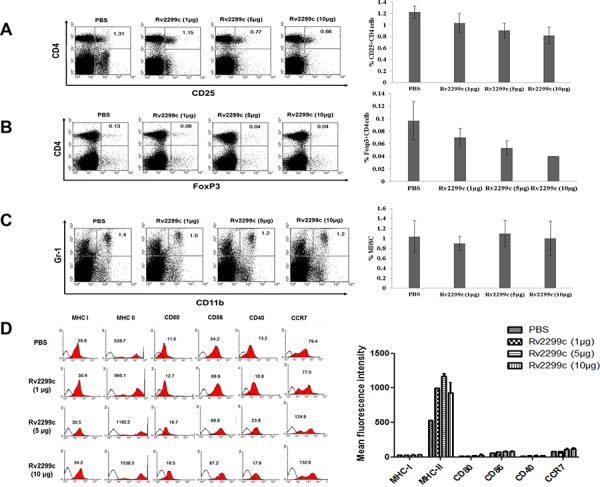
The proportions of CD4^+^ CD25^+^ regulatory T cells (Tregs), CD4^+^ Foxp3^+^ Tregs, and myeloid-derived suppressor cells (MDSCs) were measured by flow cytometry in the left panel and compared by quantitated bar graphs in the right panel **A, B.** The percentages of CD4^+^ CD25^+^ Tregs and CD4^+^ Foxp3^+^ Tregs were decreased in the 5- and 10-μg Rv2299c treatment groups compared to the 1-μg Rv2299c and PBS control groups. **C.** The percentages of MDSCs were not significantly different among the four groups. **D.** The phenotypes of DC in the spleens of tumor-bearing mice measured by flow cytometry (left panel) and compared by quantitated bar graphs in the right panel. The DC from splenocytes isolated from the mice injected with 5- and 10-μg Rv2299c showed increased expressions of MHC class I and II, CD80, CD86, CD40, and CCR7 compared to the 1-μg Rv2299c and PBS control groups. Representative histograms show marker expression (shaded) compared to the isotype controls (black line). Data are representative of more than 3 experiments.

To determine the optimal concentration of Rv2299c to achieve anti-tumor immunity against colon cancer, Rv2299c was intraperitoneally injected at doses of 1, 5, and 10 μg/mouse (Figure 2A). Treatments with 5 and 10 μg of Rv2299c showed significant inhibition of tumor growth compared to the PBS control or 1 μg of Rv2299c (*P* < 0.05) (Figure 2B; Supplemental Figure 2). To examine the tumor-specific responses, the effects of Rv2299c on the proportions among splenocytes of CD4^+^ T cells (Figure 2C), CD8^+^ T cells (Figure 2D), CD4^+^CD25^+^ Tregs (Figure 3A), CD4^+^FoxP3^+^ Tregs (Figure 3B), and CD11b^+^Gr1^+^ myeloid-derived suppressor cells (MDSCs) (Figure 3C) were evaluated in injected mice. The percentages of CD4^+^ T cells and CD8^+^ T cells increased in the 5- and 10-μg of Rv2299c treatment groups compared to the 1-μg Rv2299c group and the PBS control group. The percentages of MDSCs were not significantly different among the four groups. In contrast, the percentage of Tregs showed decreased in the 5- and 10-μg Rv2299c treatment groups compared to the 1-μg Rv2299c and PBS control groups. DC isolated from the splenocytes of mice injected with 5- and 10-μg Rv2299c showed increased expressions of MHC class I, class II, CD80, CD86, CD40, and CCR7 compared to those in the 1-μg Rv2299c and the PBS control groups (Figure 3D).

### TA-loaded DC vaccination in combination with Rv2299c treatment induced a synergistic anti-tumor immunity effect

**Figure 4:**
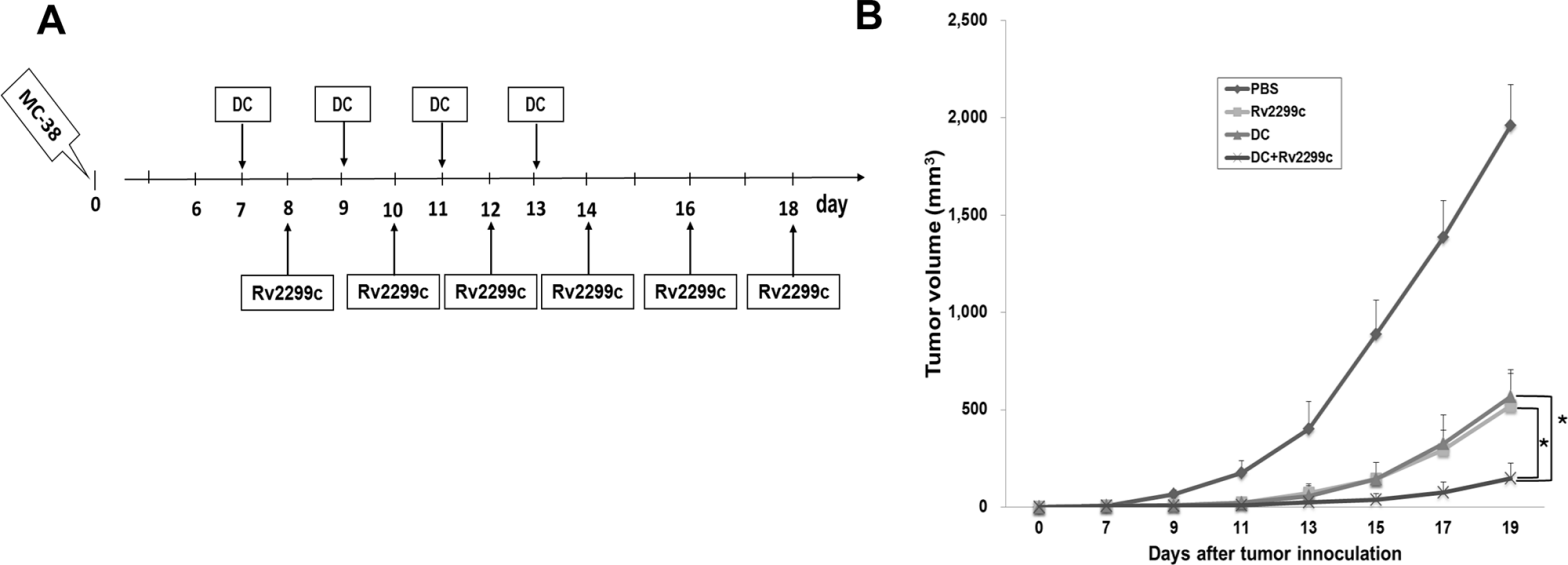
*In vivo* animal vaccination Four vaccination groups were established: 1) PBS control, 2) Tumor antigen (TA)-loaded DC vaccination, 3) Rv2299c injection, and 4) TA-loaded DC vaccination plus Rv2299c injection. On day 0, MC-38 cells (5 × 10^5^/mouse) were injected subcutaneously into the right flank of C57BL6 mice. **A.** The DC were administered subcutaneously into the left flank of C57BL6 mice on days 7, 9, 11 and 13, and Rv2299c was injected intraperitoneally on days 8, 10, 12, 14, 16 and 18. **B.** Mice vaccinated with TA-loaded DC plus Rv2299c showed significant inhibition of tumor growth (*P < 0.05 on day 19) and induced a long-term systemic anti-colon cancer immunes response (Supplemental Fig. 4) compared to TA-loaded DC or Rv2299c alone. Experiments consisted of eight mice per group.

In this study, 5-μg Rv2299c enhanced anti-tumor immunity *in vivo* more effectively than the other doses. Therefore, combination therapy of TA-loaded DC vaccine and Rv2299c was assessed for its ability to inhibit tumor growth in a colon cancer mouse model (Figure 4A). All tumor-bearing mice vaccinated with PBS showed no inhibition of the rapid growth of the tumor and had to be euthanized within three weeks. In contrast, tumor-bearing mice vaccinated with TA-loaded-DC, Rv2299c, and TA-loaded-DC plus Rv2299c showed significantly greater inhibition of tumor growth compared with the control group. Treatment with the combination of TA-loaded-DC vaccination plus Rv2299c showed significantly greater inhibition of tumor growth (*P* < 0.05) compared to TA loaded-DC or Rv2299c alone (Figure 4B; Supplemental Figure 3). Survival in mice that received a combination of TA-loaded-DC plus Rv2299c was prolonged compared to that of mice that received the TA-loaded-DC or Rv2299c alone (Supplemental Figure 4). These results indicated that TA-loaded DC vaccination plus Rv2299c induced a long-term systemic anti-colon cancer immune response in a mouse model.

### Activation of CTLs and NK cells by vaccination with TA-loaded DC plus Rv2299c

**Figure 5:**
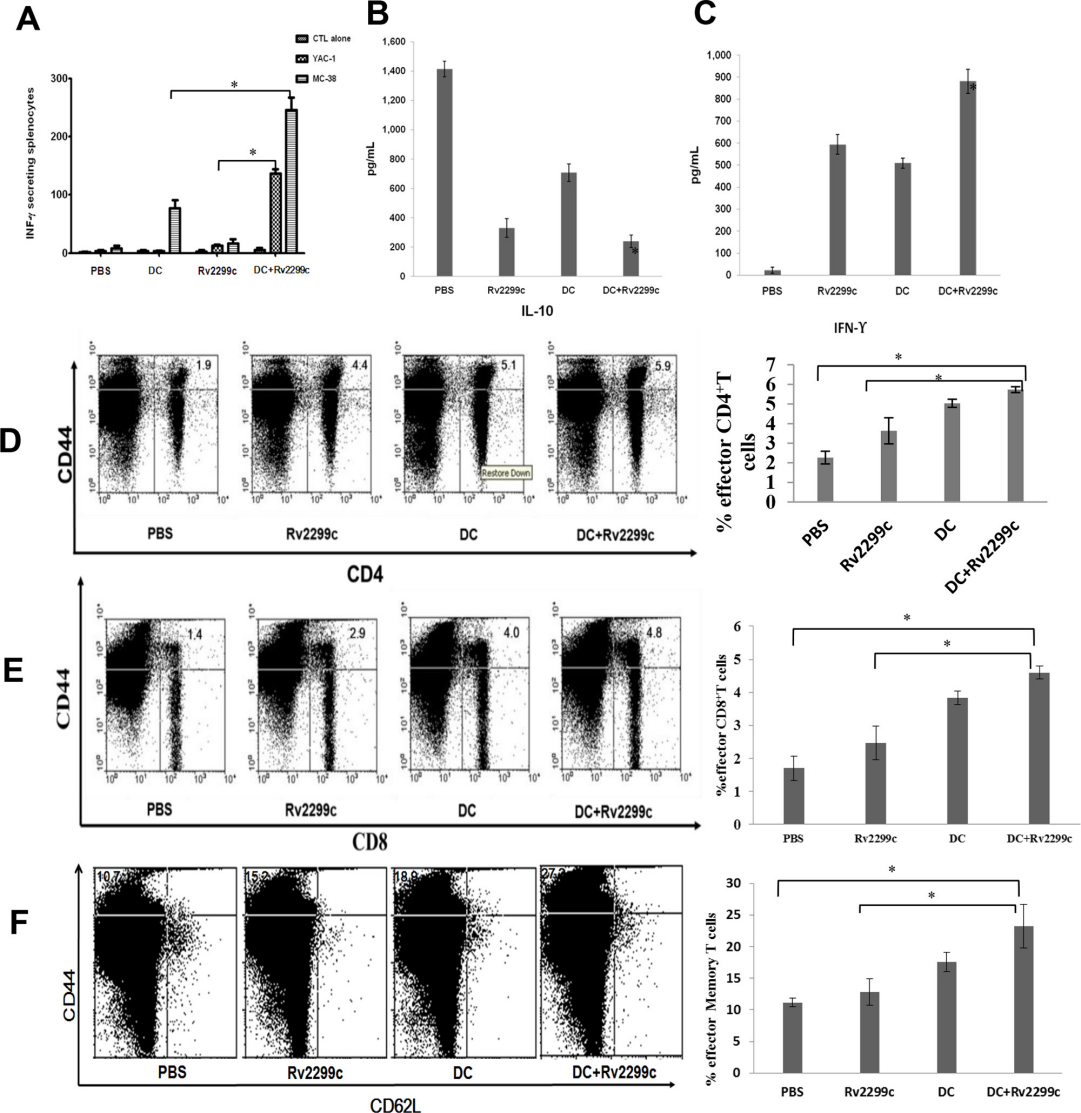
Activation of cytotoxic T lymphocytes (CTLs) and natural killer (NK) cells, the proportion of CD4^+^ T cells, CD8^+^ T cells, memory T cells, and cytokine production induced by vaccination with TA-loaded DC plus Rv2299c **A.** The number of IFN-γ-secreting lymphocytes in spleens of mice treated with PBS, TA-loaded DC, Rv2299c and DC plus Rv2299c were counted in an IFN-γ ELISPOT assay. A combination of DC vaccination and Rv2299c injection significantly increased the number of IFN-γ-secreting lymphocytes against MC-38 and YAC-1 cells compared to other groups (*P < 0.05). **B.** IL-10 and **C.** IFN-γ production from splenocytes of vaccinated mice was evaluated by ELISA. The culture supernatants of splenocytes from the mice vaccinated with TA-loaded DC plus Rv2299c showed lower level of IL-10 and higher level of IFN-γ compared to other mice (*P < 0.05). Data are shown as means (pg/mL) ± SD of triplicate cultures from three independent experiments. The proportions of **D.** CD4^+^ T cells, **E.** CD8^+^ T cells, and **F.** memory T cells were measured by flow cytometry in the left panel and compared by quantitated bar graphs in the right panel. The results revealed that there were significant increases of effector cells in the TA-loaded DC groups with or without Rv2299c compared to the group with Rv2299c alone. The data are representative of more than 3 experiments. Statistical comparisons were performed using the one-way nonparametric ANOVA.

To investigate the anti-tumor effect of CTLs produced by TA-loaded DC vaccination in a mouse MC-38 colon cancer model, splenocytes from each group of vaccinated mice were prepared for IFN-γ ELISPOT assays. MC-38 and YAC-1 cells were used as the target cells. In comparison with PBS control, vaccination with Rv2299c alone did not increase the number of IFN-γ-secreting splenocytes against MC-38 cells, whereas vaccination with TA-loaded DC or TA-loaded DC plus Rv2299c led to a significant increase in IFN-γ-secreting splenocytes against MC-38 cells (*P* < 0.05). In addition, TA-loaded DC plus Rv2299c vaccination showed the highest number of IFN-γ-secreting splenocytes against MC-38 cells among other groups. The cytotoxicity of NK cells (shown by the number of IFN-γ-secreting splenocytes against YAC-1 cells) was significantly higher in the two groups of mice injected with Rv2299c than those groups not injected with Rv2299c. Compared to Rv2299c treatment alone, TA-loaded DC plus Rv2299c injection induced superior NK cell activity against the target cells (*P* < 0.05; Figure 5A). These results indicated that the tumor-inhibitory effects of TA-loaded DC plus Rv2299c injection were resulted from the CTL-mediated responses induced by TA-loaded DC vaccination and the NK cells-mediated responses induced by the Rv2299c injection. In this study, vaccination with TA-loaded DC plus Rv2299c led to production of lower levels of IL-10 compared to the PBS control, TA-loaded DC alone, or Rv2299c alone (Figure 5B). In contrast, IFN-γ production by TA-loaded DC plus Rv2299c was higher compared to that by TA-loaded DC or Rv2299c alone (Figure 5C). These results suggested that the combination of TA-loaded DC plus Rv2299c vaccination enhanced Th1 responses in addition to tumor-specific CTL responses. Regarding tumor-specific responses, the percentages of CD4^+^ T cells (Figure 5D), CD8^+^ T cells (Figure 5E), and memory T cells (Figure 5F) were increase significantly in the TA-loaded-DC vaccination groups compared to the group administered Rv2299c alone.

### Efficient inhibition of MDSCs and Tregs by the combination of TA-loaded DC vaccination plus Rv2299c treatment

**Figure 6:**
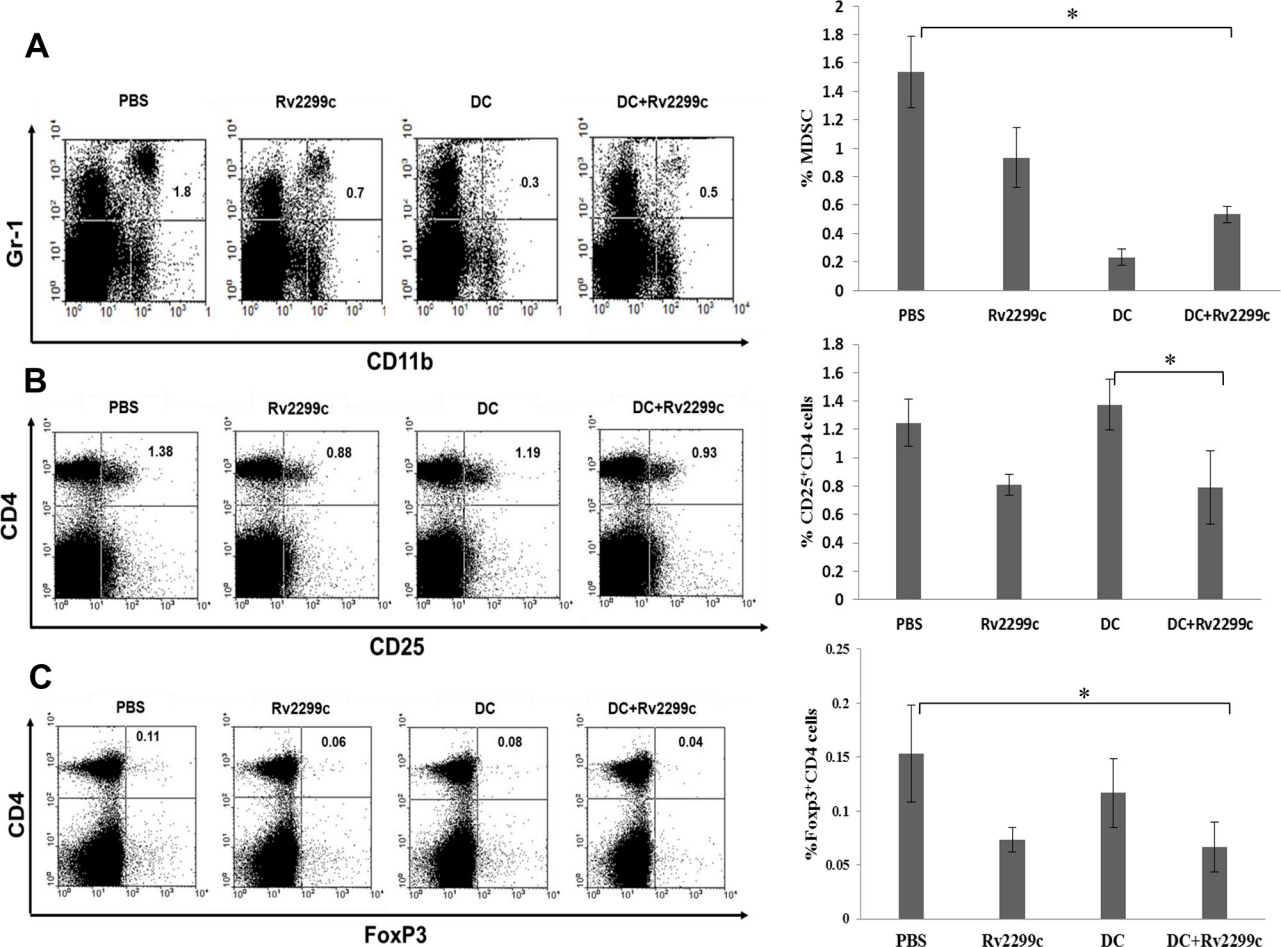
The proportions of **A.** MDSCs, **B.** CD4^+^ CD25^+^ Tregs and **C.** CD4^+^ Foxp3^+^ Tregs in the spleens of vaccinated tumor-bearing mice were measured by flow cytometry in the left panel and compared by quantitated bar graphs in the right panel. The proportions of MDSCs and Tregs were significantly increased in the PBS control group after tumor inoculation. Rv2299c alone increased the generation of MDSCs compared to TA-loaded DC alone or TA-loaded DC plus Rv2299c, while the percentages of Tregs were lower in the Rv2299c adjuvant and TA-loaded DC plus Rv2299c groups compared to the TA-loaded DC alone group. And the data are representative of more than 3 experiments. Statistical comparisons were performed using the one-way nonparametric ANOVA (*P < 0.05).

To investigate the immunological mechanisms underlying the enhanced tumor-specific immune responses, the effects of the combination therapy on the proportion of MDSCs (CD11b^+^Gr1^+^) and Tregs (CD4^+^FoxP3^+^ and CD4^+^FoxP3^+^ cells) were analyzed. The percentages of MDSCs (Figure 6A) and Tregs (Figures 6B and 6C) were significantly increased in the PBS control group after tumor inoculation. Administration of Rv2299c alone increased the generation of MDSCs compared to TA-loaded-DC alone or TA-loaded-DC plus Rv2299c, while the percentages of Tregs decreased in the Rv2299c adjuvant and TA-loaded-DC plus Rv2299c compared to TA-loaded-DC alone. These findings suggested that the combination of TA-loaded DC plus Rv2299c vaccination enhanced therapeutic anti-tumor immunity by inhibiting the immunosuppressive tumor microenvironment during the vaccination phases.

## DISCUSSION

In this study, we demonstrated that the TLR-4 agonist Rv2299c could be used as a potential adjuvant with TA-loaded DC in a colon cancer vaccine. Local injection of Rv2299c together with a TA-loaded DC vaccine resulted in significantly greater protection from the progression of colon cancer than either therapy alone. Additionally, Rv2299c enhanced the priming of tumor-specific CD8^+^ T cells. Thus, we considered that the use of a TLR agonist, such as Rv2299c, may act as potent innate immune response modifier that can enhance the tumor microenvironment and “danger signals” critical for generating potent Th1-type immune response and effective antitumor immunity [21]. Our finding also highlights the delicate nature of immune responses in a mouse colon cancer model. Administration of Rv2299c resulted in a dramatic decrease in tumor growth, and we believe that the Rv2299c administration may still efficacious without inducing mortality in this model.

Recent evidence suggests that persistent TLR signaling may assist in bypassing regulatory T cell-induced immune tolerance [22], enhancing autoimmune T cell responsiveness [23], or even reversing regulatory T cell function [24]. These effects may represent an additional mechanism underlying the synergistic effect of the TLR-4 agonist with DC immunotherapy.

In conclusion, our data suggest that TA-loaded DC vaccination with Rv2299c derived from mycobacteria enhanced anti-tumor immunity in a mouse colon cancer model by inhibiting immunosuppressive cells during tumor progression. This, in turn, enhances the anticancer effects of vaccination, triggering direct co-stimulatory effects, including the proliferation and activation of T cells, and the subsequent generation of Th1-type immunity with enhancement of effector NK cell function. Both properties of Rv2299c should prove useful in a clinical context; it is known that Rv2299c exerts general immunostimulatory effects in colon cancer patients. Future studies are needed to determine the exact mechanisms by which TLR agonists enhance DC immunotherapy, and to understand how various TLR agonists can be used safely in colon cancer immunotherapy.

## MATERIALS AND METHODS

### Mice, tumor cell lines and Rv2299c

Six- to eight-week-old female C57BL6 mice were purchased from Orient Bio (Iksan, Republic of Korea), and were maintained under specific pathogen-free conditions. All animal care, experiments and euthanasia were performed in accordance with protocols approved by the Chonnam National University Animal Research Committee. Murine MC38 colon cancer cell and YAC-1 cell lines were purchased from the American Type Culture Collection (Rockville, MD, USA). All cell lines were maintained in Dulbecco's Modified Eagle's medium (DMEM: Gibco-BRL, Grand Island, NY, USA) supplemented with 10% fetal bovine serum (FBS) (Gibco-BRL) and 1% penicillin/streptomycin (PS). Rv2299c was kindly provided by professor Sung-Jea Shin (Yonsei University College of Medicine, Republic of Korea). For *in vivo* mouse injections, Rv2299c stock solutions were diluted in sterile 0.9% normal saline.

### Generation of bone-marrow (BM)-derived DC

C57BL6 BM-derived immature DC (iDC) were generated as described previously [25]. Briefly, BM was harvested from the femurs and tibiae of mice and cultured in RPMI-1640 (Gibco-BRL) supplemented with 10% FBS (Gibco-BRL) and 1% PS, in the presence of 10 ng/mL recombinant murine (rm) GM-CSF (R&D Systems, Minneapolis, MN, USA) and 10 ng/mL rmIL-4 (R&D Systems). On days 2 and 4, the medium and cytokines were replaced with fresh complete medium-containing cytokines. On day 6, iDC were purified by positive selection with CD11c^+^-magnetic beads (Miltenyi Biotec, Auburn, CA, USA). Mature DC were then generated by a further 48-h cultivation of CD11c^+^ DC with 10 ng/mL rmTNF-α (R&D systems), 10 ng/mL rmIL-1β (R&D systems) and 10 ng/mL rmGM-CSF (R&D systems).

### Generation of tumor antigen-loaded DC

Apoptotic cells were induced by γ-irradiation (100 Gy) (Gammacell-1000 Elite, MDS Nordion, Canada) followed by overnight culturing in RPMI-1640 without FBS. The apoptotic γ-irradiated cells were confirmed using an Annexin V-FITC Apoptosis Detection Kit (BD Bioscience, San Jose, CA, USA) and loaded on iDC at 2 h after the addition of maturating cytokines at a ratio of 2:1 (DC:apoptotic cells).

### *In vivo* animal vaccination

Four vaccination groups were established: 1) PBS control, 2) TA-loaded DC vaccination, 3) Rv2299c injection, and 4) TA-loaded DC vaccination plus Rv2299c injection. On day 0, mice were injected subcutaneously with 5 × 10^5^ MC38 cells in the right flank in a volume of 0.1 mL. TA-loaded DC vaccine (1 × 10^6^ cells/mouse) was administered subcutaneously in the left flank of C57BL6 mice in a volume of 0.1 mL of PBS at 2-day interval on days 7, 9, 11 and 13, and Rv2299c (5 μg/mouse) was injected intraperitoneally in a volume of 0.1 mL on days 8, 10, 12, 14, 16 and 18. To assess the anti-tumor efficacy of the vaccinated mice, three perpendicular dimensions (length, width and height) of each tumor were measured individually every 2 days using a Vernier caliper, and the tumor volume was calculated using the following formula for the standard volume of an ellipsoid: *V* = 4/3 π (length × width × height/8).

### Phenotype analysis of splenocytes from vaccinated mice

At the indicated time points, the mice were sacrificed and splenocyte phenotypes were characterized by their cell surface markers using fluorescently labeled monoclonal antibodies (mAbs) and analyzed by flow cytometry. The cells were stained with the following mAbs (eBioscience, San Diego, CA, USA): CD11b-FITC, CD11c-PE, Gr-1-PE, CD4-PE, FOXP3-Alexa Fluor, CD25-FITC, CD3-FITC, CD8-PE, CD44-PE, and CD62L-FITC. Isotype-matched controls were run in parallel. Cell debris was eliminated by forward and side scatter gating. The samples were acquired on a FACS Calibur cell sorter (Becton Dickinson, Mountain view, CA, USA) and the data was analyzed using WinMDI Version 2.9 software (Biology Software Net: http://en.bio-soft.net/other/WinMDI.html).

### TA-specific CTL activities of vaccinated mice

Splenocytes (1 × 10^6^ cells) isolated from vaccinated mice 7 days after the final immunization (day 20) were added to 24-well plates and restimulated with γ-irradiated MC38 cells (5 × 10^5^ cells) for 5 days in RPMI-1640 (Gibco-BRL) containing 10% FBS (Gibco-BRL) and 1% PS supplemented with 20 ng/mL rmIL-2 (R&D systems) is better. After stimulation, the splenocytes were assessed for TA-specific CTLs using a mouse IFN-γ Enzyme-Linked Immunospot (ELISPOT) assay (BD Bioscience). The MC38 cell line and NK-sensitive YAC-1 cell line were used as target cells.

### *In vitro* analysis of cytokine production from vaccinated mice

Cytokine (IFN-γ and IL-10) production from vaccinated mice was determined by a BD OptEIA™ enzyme-linked immunosorbent (ELISA) assay (BD Bioscience). Supernatant from the stimulated splenocytes of all vaccinated mice was used for measurement of the production of Th1- and Th2-polarizing cytokines. Each sample was analyzed in triplicate and the mean absorbance for each set of standards and samples was calculated.

### Intracellular staining assay of regulatory T cell (Treg) generation in the spleens of vaccinated mice

To evaluate the proportion of Tregs, 1 × 10^6^ splenocytes from vaccinated mice were harvested and stained with PE-conjugated CD4 and FITC-conjugated CD25 mAbs for 30 min at 4°C. Fc block was added before incubation with surface antibodies. The cells were then washed and permeabilized with FACSTM Permeabilizing Solution 2 (BD Bioscience) for 30 min at room temperature. After washing twice, the cells were stained with an Alexa Fluor -conjugated Foxp3 antibody (Miltenyi Biotec) for 30 min at 4°C. The samples were acquired on a FACS Calibur cell sorter (Becton Dickinson) and the data were analyzed using WinMDI Version 2.9 software (Biology software Net).

### Statistical analysis

The Mann-Whitney *U* test was performed to assess the significance of non-parametric differences among the groups. Survival of the vaccinated mice was analyzed using SigmaPlot 10.0 (Systat Software Inc. San Jose, CA, USA). *P* values <0.05 were considered to indicate statistical significance.

## SUPPLEMENTARY FIGURES


