# Supplementary material for: Dendritic cell vaccination with a toll-like receptor agonist derived from mycobacteria enhances anti-tumor immunity
Source: Oncotarget. 2015 Sep 16;6(32):33781–90. doi: 10.18632/oncotarget.5281 (PMC4741802; doi:10.18632/oncotarget.5281)
Supplement: Supplementary file 1 [file oncotarget-06-33781-s001.pdf]

SUPPLEMENTARY FIGURES

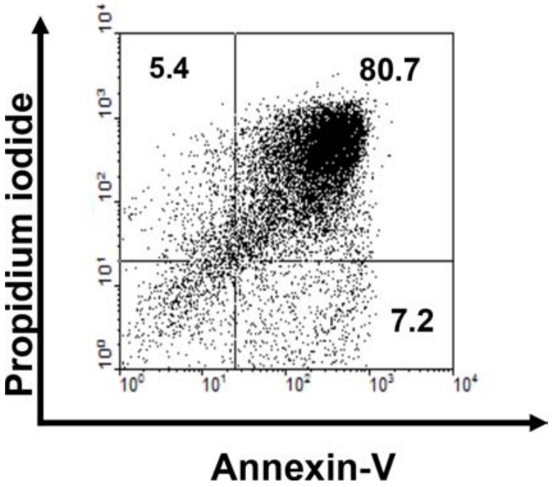

Supplementary Figure S1: Apoptosis in the γ-irradiated MC-38 cells was confirmed by staining with annexin-V and propidium iodide, ~96% of cells underwent apoptosis.

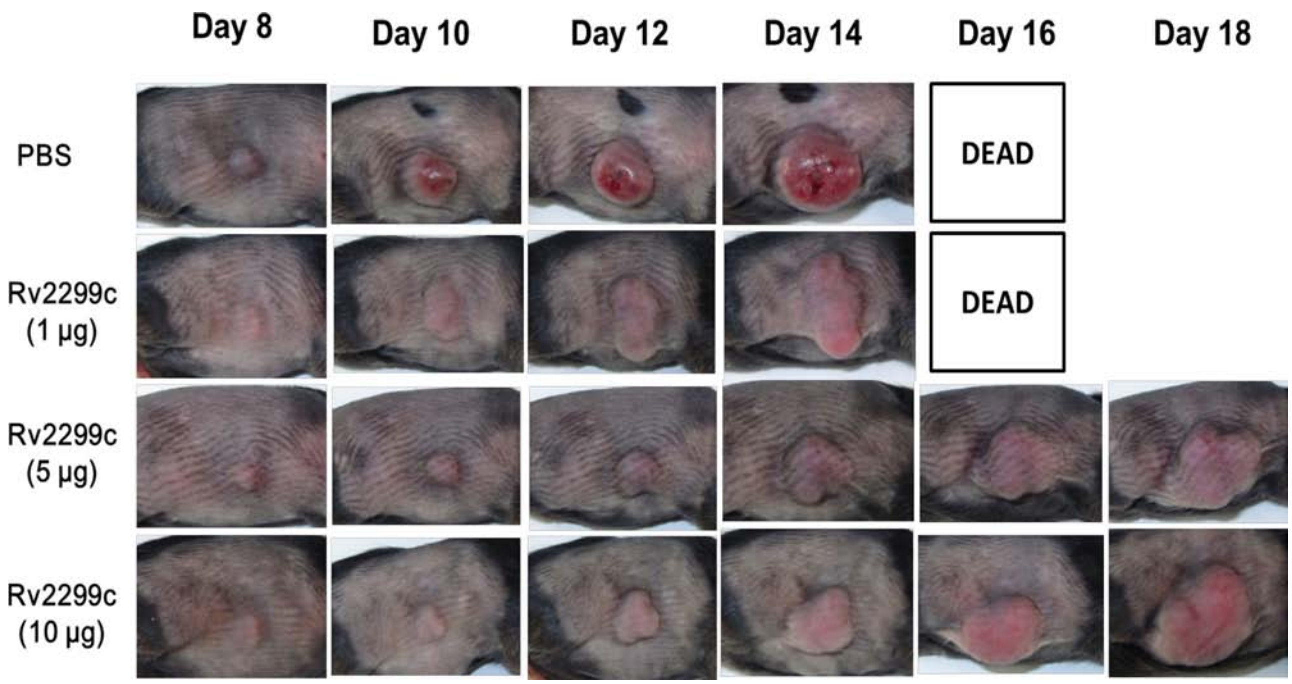

Supplementary Figure S2: Representative pictures of mice treated with 5- and 10-μg Rv2299c showed significantly greater inhibition of tumor growth compared to those receiving 1-μg Rv2299c or the PBS control ($P < 0.05$).

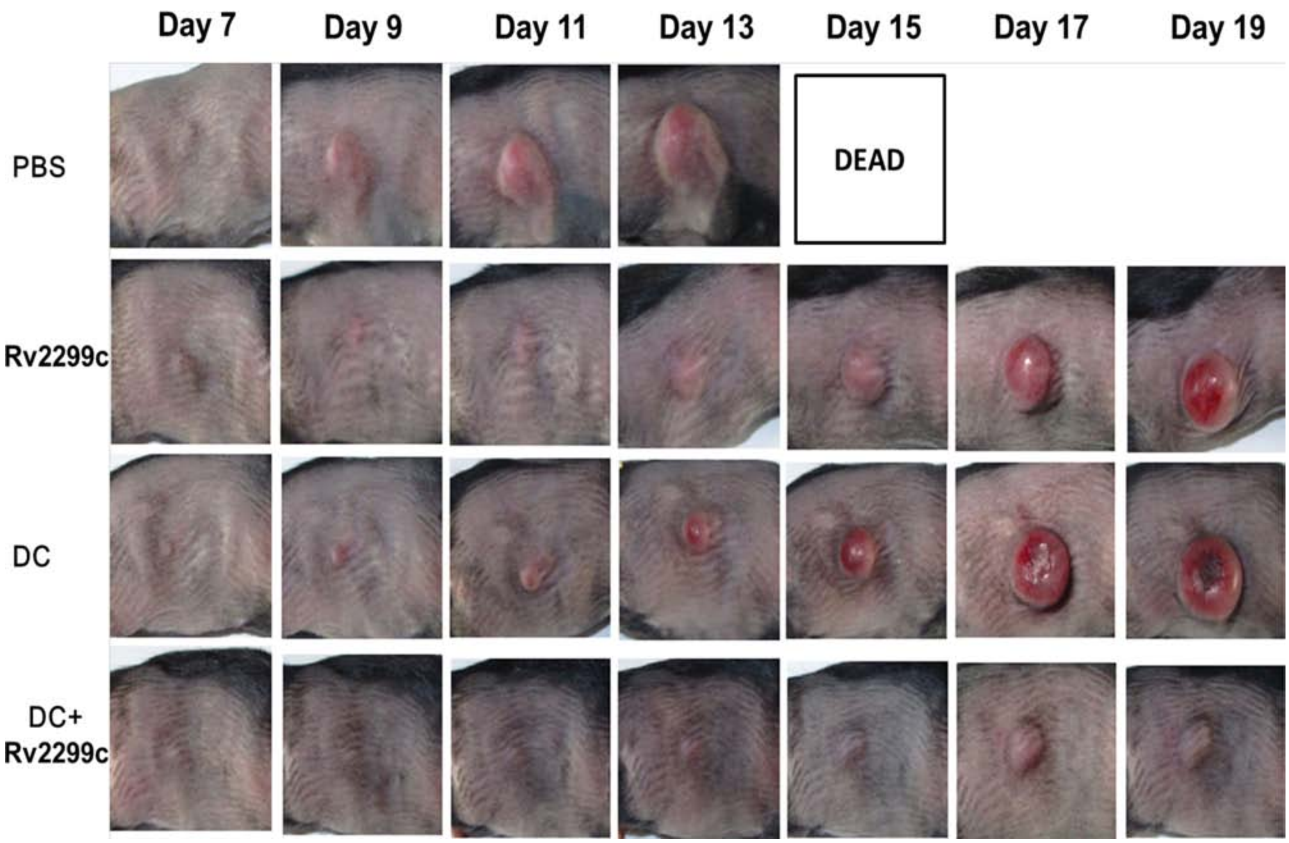

Supplementary Figure S3: Representative pictures of mice vaccinated with TA-loaded DC plus Rv2299c showed significant inhibition of tumor growth compared to the PBS control or TA-loaded DC or Rv2299c injection (P < 0.05 on day 19). Experiments consisted of eight mice per group.

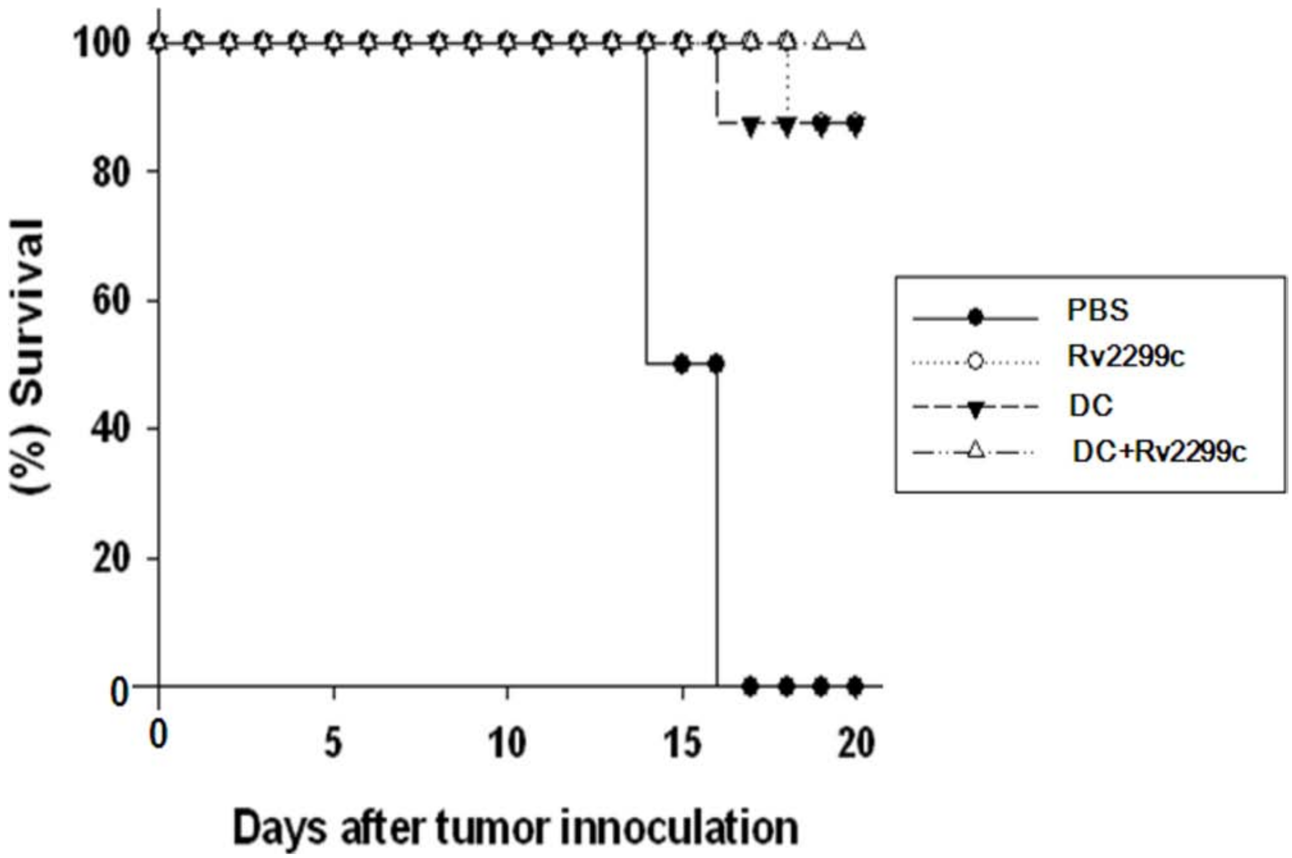

Supplementary Figure S4: The combination of TA-loaded DC vaccination plus Rv2299c injection prolonged survival of vaccinated mice. The survival rate of the vaccination groups was evaluated every 2 days. Experiments consisted of eight mice per group.
